# Modulation of Oxidative Stress by Twist Oncoproteins

**DOI:** 10.1371/journal.pone.0072490

**Published:** 2013-08-13

**Authors:** Nicolas Floc'h, Jakub Kolodziejski, Leila Akkari, Yannick Simonin, Stéphane Ansieau, Alain Puisieux, Urszula Hibner, Patrice Lassus

**Affiliations:** 1 Institut de Génétique Moléculaire de Montpellier, Centre National de la Recherche Scientifique (CNRS), Unité Mixte de Recherche (UMR) 5535, Montpellier, France; 2 Université Montpellier I and Université Montpellier II, Montpellier, France; 3 Institut National de la Santé et de la Recherche Médicale (Inserm) Unité Mixte de Recherche (UMR) S1052, Centre de Recherche en Cancérologie, Lyon, France; 4 Centre National de la Recherche Scientifique (CNRS), Unité Mixte de Recherche (UMR) 5286, Centre de Recherche en Cancérologie, Lyon, France; 5 Université Unité Mixte de Recherche (UMR) 1052, Centre de Recherche en Cancérologie, Lyon, France; 6 Université de Lyon, Lyon, France; 7 Centre Léon Bérard, Lyon, France; Univ. Paris Diderot, Sorbonne Paris Cité, France

## Abstract

Expression of developmental genes Twist1 and Twist2 is reactivated in many human tumors. Among their oncogenic activities, induction of epithelial to mesenchymal transition is believed to increase cell motility and invasiveness and may be related to acquisition of cancer stem cell phenotype. In addition, Twist proteins promote malignant conversion by overriding two oncogene-induced failsafe programs: senescence and apoptosis. Reactive oxygen species (ROS) are also important mediators of apoptosis, senescence and motility and are tightly linked to disease, notably to cancer. We report here that Twist factors and ROS are functionally linked. In wild type cells both Twist1 and Twist2 exhibit antioxidant properties. We show that Twist-driven modulation of oncogene-induced apoptosis is linked to its effects on oxidative stress. Finally, we identify several targets that mediate Twist antioxidant activity. These findings unveil a new function of Twist factors that could be important in explaining their pleiotropic role during carcinogenesis.

## Introduction

The two Twist genes (Twist1 and Twist2), related in sequence and function, belong to the super-family of basic Helix-Loop-Helix (bHLH) transcription factors. They are essential for embryonic development as they are involved in mesoderm patterning and the differentiation of multiple cell lineages, including muscle, cartilage and osteogenic cells [1]. Their embryonic functions appear to be related to their ability to induce cell migration by promoting the epithelial to mesenchymal transition (EMT) [2], to protect cells from apoptosis [3] and to control inflammation [4]. Twist expression was reported in several types of precursors and stem cells [5], [6], where it was suggested to control the maintenance of their pluripotency. In contrast, Twist proteins are not detectable in most differentiated tissues [7], but their expression is reactivated in many human cancers, including carcinomas (breast, liver, ovarian, prostate), sarcomas, melanomas, gliomas and neuroblastomas [8]. Their oncogenic potential is thought to arise from a combination of multiple properties. First, by promoting the epithelial to mesenchymal transition (EMT), Twist proteins favour invasiveness and may confer to cells self-renewal properties [9], [10], [11], [12]. Second, by disrupting both Rb- and p53-dependent pathways, they override the two major oncogene-induced cellular failsafe programs, which are senescence and apoptosis [13], [14], [15], thereby promoting malignant conversion. Third, several reports describe a role for Twist1 in the acquisition of resistance to therapeutic drugs [16], [17], linking Twist to tumor recurrence.

Reactive oxygen species (ROS), which include superoxide anions, H_2_O_2_, organic peroxides, and hydroxyl radicals, are by-products of oxidative phosphorylation and as such are constantly generated in all aerobic cells. ROS are eliminated by antioxidant enzymes: scavenger enzymes, superoxide dismutases (SOD), convert superoxide anions to H_2_O_2_, which is subsequently detoxified by catalase, glutathione peroxidase or thioredoxin peroxidase systems [18]. ROS, although essential for processes such as immune response, angiogenesis and stem cell renewal [19], can also be harmful for cells. An uncontrolled production of these molecules provokes damage, including DNA lesion, protein oxidation, and lipid peroxidation [19]. Cellular response to major unrepaired damage includes apoptosis or senescence. Because Twist and ROS overproduction both impact on activities related to the regulation of several common cellular processes [13], [15], we explored the possibility that Twist is involved in the control of ROS and, more generally, in cellular response to oxidative stress.

We report here that Twist lowers the level of intracellular ROS, displaying an antioxidant activity in several cell types. Twist-driven inhibition of oxidative stress participates in the protection of cells against c-Myc induced apoptosis. Analysis of molecular mechanisms involved in this new function reveals the existence of an oxidative genetic program mediated by several new targets. Our findings unveil a novel property of Twist proteins that could have important implications for their role during tumor progression.

## Results

### Twist proteins display antioxidant properties

**Figure 1:**
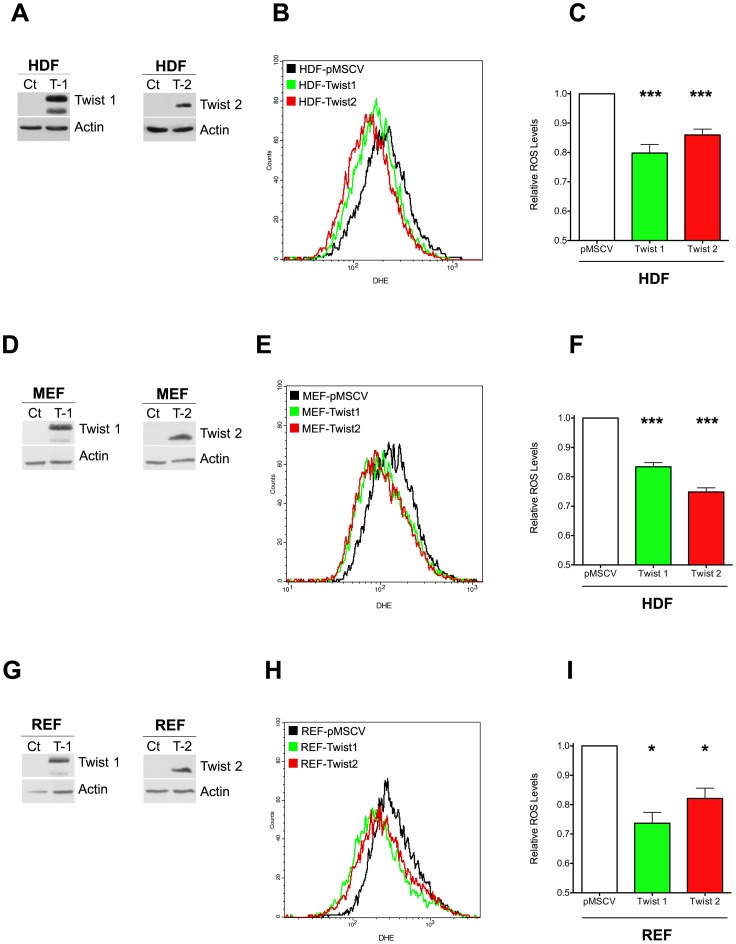
Twist proteins lower intracellular ROS levels. (A–C) HDF, (D–F) MEF and (G–I) REF52 cells were transduced with retroviruses encoding either Twist1 (T–1) or Twist2 (T–2) tagged with a Myc epitope. The empty vector (pMSCV) was used as a control. (A, D and G) Expression of the transgenes was verified by western-blotting (CT:pMSCV; T-1:Twist1, T-2:Twist2). (B, E and H) ROS levels were analyzed by flow cytometry after DHE labeling. (C, F, and I) Quantitative analysis of ROS levels in HDF (C), MEF (F) and REF52 (I) cells with data representing the mean values ± SEM of the fluorescence geometric means (HDF: n = 10; MEF: n =  8; REF: n = 3, *: compared to control).

To investigate the effect of Twist proteins on ROS production, we first generated foreskin-derived human diploid fibroblasts (HDF) ectopically expressing Twist1 or Twist2. The cells displayed no noticeable morphological changes and grew at a similar rate to both parental and control cells (data not shown). As assessed by labeling cells with DiHydroEthidium (DHE) (Figure 1A–C) or with DiChlorodihydroFluorescein DiAcetate (DCFDA) (Figure S1A–B), two ROS fluorescent indicators, ectopic expression of either Twist1 or Twist2 was associated with a significant reduction of intracellular ROS. Expression of Twist was as efficient in inhibiting ROS as was the treatment of cells with 10 mM N-acetyl-cysteine (NAC), a widely used antioxidant compound (Figure S1C–D). The antioxidant property of both Twist proteins was next confirmed using additional cellular models, including primary Mouse Embryonic Fibroblasts (MEFs) (Figure 1D–F), immortalized Rat Embryonic Fibroblasts (REF52) (Figure 1G–I) and immortalized human mammary epithelial cells (HMEC-hTert) (Figure S1E). These results indicate that the antioxidant activity of Twist is neither cell type- nor species-dependent.

**Figure 2:**
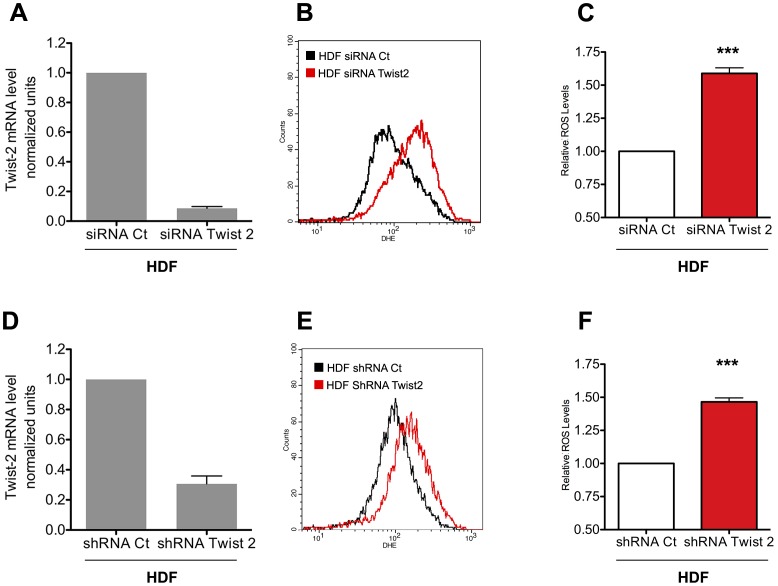
Twist2 inhibition increases oxidative stress. (A) HDF cells were transfected with a siRNA control or a siRNA directed against Twist2 and the silencing efficiency was measured by RT-qPCR normalized to GAPDH. (B) The level of ROS in control and Twist2-inhibited HDF cells was monitored by labeling with DHE and a representative FACS histogram is shown. (C) ROS levels are presented as mean geometric fluorescence intensities (± SEM) in cells transfected with a siRNA against Twist2 relative to a control siRNA (*: n = 6, relative to control). (D) HDF were infected with a retroviral vector (pRetro-SUPER-puromycin) containing a shRNA sequence directed against Twist2 or an irrelevant sequence as a control, selected and analyzed 3 to 5 days post-selection. Knockdown efficiency was measured by RT-qPCR relative to GAPDH. (E) DHE fluorescence was analyzed as in panel B. (F) Quantitative analysis of ROS was determined as in C (*:n = 6, relative to control).

We reasoned that since ectopic expression of Twist diminished ROS levels, inhibition of endogenous Twist expression should give rise to the opposite effect. Since HDFs mainly express Twist2 (data not shown), we explored the consequences of its depletion in these cells. This was achieved through the use of either siRNA or shRNA that recognized unrelated sequences of Twist2 mRNA. Both gave rise to inhibition of Twist2 expression and, in accord with our hypothesis, both led to a significant increase of endogenous ROS (Figure 2). Of note, this increase was reverted by NAC (Figure S2). Overall, our results strongly suggest that Twist proteins exhibit antioxidant properties in several cellular models.

### Twist, ROS and c-Myc-induced apoptosis

As ROS drive induction of the same cellular failsafe programs [20], [21] that are overridden by Twist proteins, we assessed whether the newly identified antioxidant property of Twist had an impact on its oncogenic potential. In light of the role of ROS in c-Myc induced cell death and transformation [21], [22], [23], we focused our attention on Twist's anti-apoptotic activity, which was originally discovered through a genetic screen aimed at identifying genes that counteracted c-Myc induced apoptosis [14]. Interestingly, several subsequent reports showed that generation of ROS accompanied c-Myc overexpression and was linked to the cell death phenotype, as well as to DNA damage, genetic instability and cellular transformation [21], [22], [23].

**Figure 3:**
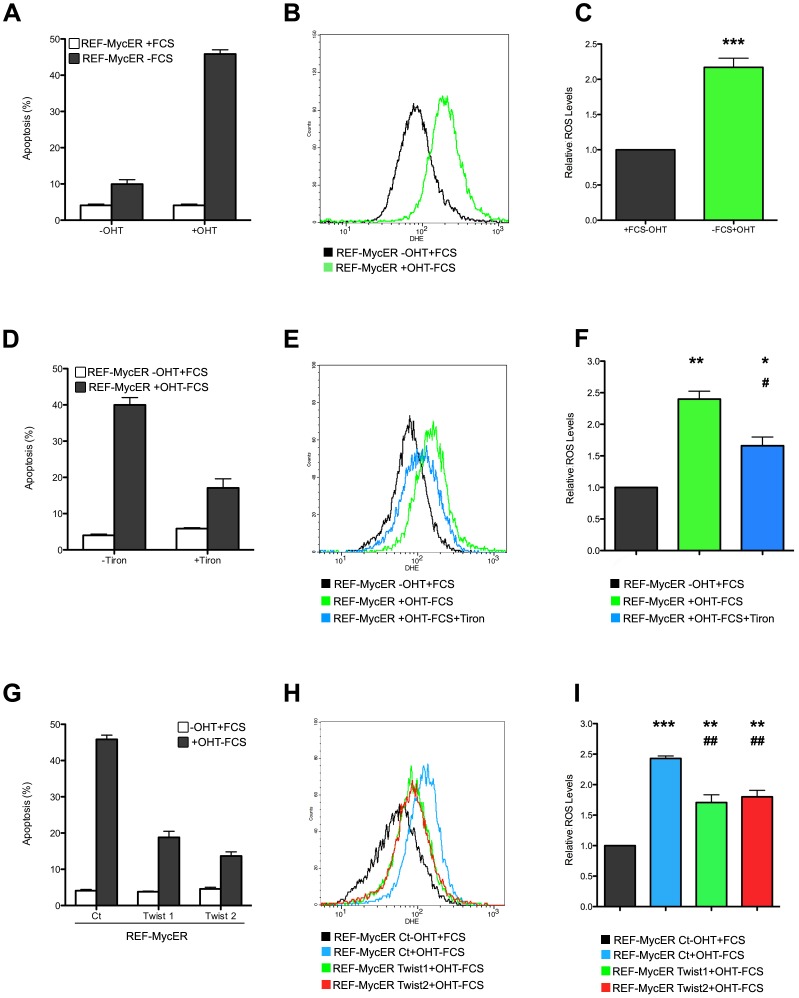
Twist inhibits c-Myc-induced ROS accumulation and apoptosis. (A) REF52 cells expressing a MycER construct were cultured for 24 hours in 0.1% serum (dark bars) in the presence or absence of 4-hydroxytamoxifen (OHT), as indicated, to induce c-Myc activity. Control cells were cultured in 10% serum (white bars). Cells were collected, labelled with Cy3-Annexin-V and apoptosis was quantified by flow cytometry. Error bars represent mean ± SEM of three independent experiments. (B) REF52-MycER cells were treated for 8 hours as in A and DHE fluorescence was measured as in Figure 1B. (C) Represents the quantitative analysis of B (*: n = 7, compared to control). (D) REF52-MycER cells were cultured in the presence of 10% FBS and absence of OHT (white bars) or in 0.1% serum with OHT (dark bars) in the presence or absence of 2 mM Tiron, as indicated. Apoptosis was quantified as in A. (E) Cells were cultured as in D and ROS levels were monitored as in B. (F) Quantification of E (*: n = 6, compared to black bar. ^#^: n = 6, compared to green bar). (G) Quantification of apoptosis in REF52-MycER cells expressing Twist1 or Twist2. Black bars: cells are grown under pro-apoptotic conditions (active MycER, 0.1% FBS), white bars: control cultures. Cell death was quantified by Cy3-Annexin-V staining as in A. (H) Cells expressing Twist1 or Twist2 were treated as in G and ROS levels were determined as in D. (I), quantification of ROS levels from H (*: n = 6, compared to black bar. ^#^: n = 6, compared to blue bar).

To investigate whether the anti-apoptotic function of Twist is functionally related to its antioxidant properties, we ectopically expressed a tamoxifen inducible c-Myc-ER fusion construct in rat embryonic fibroblasts (REF52), the cell type that was used to unveil the apoptotic activity of c-Myc [24]. We first confirmed that c-Myc activation sensitized REF52 cells to apoptosis upon growth factor withdrawal (Figure 3A). Interestingly, these conditions are associated with a significant increase in endogenous ROS production (Figure 3B–C) provoked mainly by the removal of serum (Figure S3A). To evaluate whether ROS production is instrumental in c-Myc induced cell death in this model, cells were cultured in the presence of Tiron, an efficient antioxidant compound [25]. Of note, unlike NAC, Tiron did not display any cytotoxic effects in REF52. As shown in Figure 3D–F, reduction of ROS by this antioxidant compound was associated with improved cell survival, confirming the reported role played by ROS in c-Myc-induced apoptosis [26]. As expected, we found that ROS induction was necessary but not sufficient to induce a strong apoptotic response in this model (Figure S3B).

**Figure 4:**
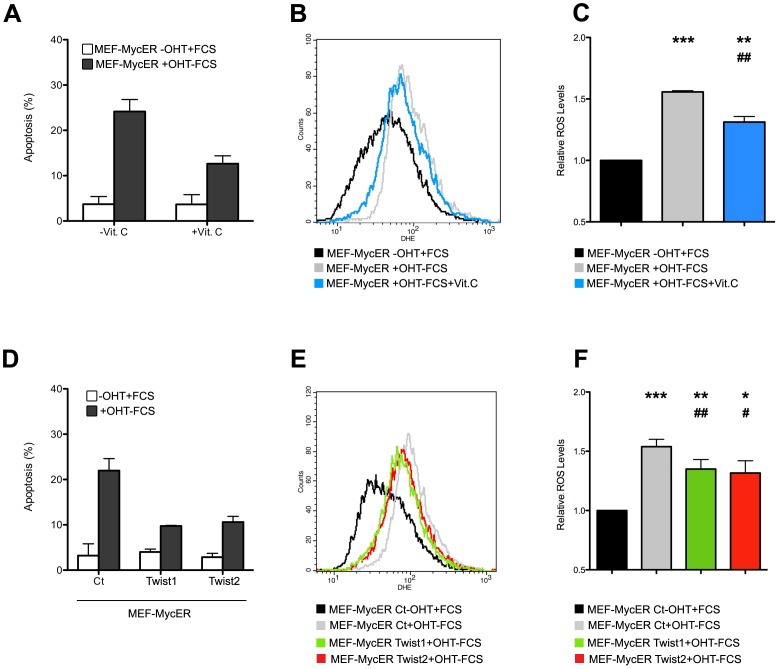
Twist antioxidant activity is associated with modulation of apoptosis in primary mouse embryonic fibroblasts. (A) MEF cells expressing the MycER construct were cultured in the presence of 0.5% or 10% FBS, with or without OHT, and in the presence or absence of Vitamin C, used as an antioxidant. Apoptosis was quantified by Annexin-V staining and is presented as means ± SEM of three independent experiments. (B) MEF-MycER cells were treated as in A and ROS was measured by DHE staining by flow cytometry. (C) Quantification of B (*: n = 5, compared to black bar. ^#^: n = 5, compared to grey bar). (D) MEF-MycER cells were infected with either Twist1, Twist2 or pMSCV vectors and selected with puromycin. Cells were then cultured for 24 hr. in 10% FBS in the absence of OHT (white bars) or in 0.5% FBS supplemented with OHT (dark bars). Apoptosis was determined as in A. (E) ROS levels in all cell lines was monitored as in B. (F) Quantitative analysis of E (*: n = 6, compared to black bar. ^#^: n = 6, compared to grey bar).

As previously described, ectopic expression of Twist proteins protected cells from c-Myc-induced apoptosis [14] upon growth factor withdrawal (Figure 3G). Strikingly, the increased survival was associated with a significant reduction in ROS (Figure 3H–I, Figure S3A), suggesting that the antioxidant property of Twist is indeed related to its oncogenic potential. Comparable results were obtained in experiments performed using mouse embryonic fibroblasts (MEFs) (Figure 4).

We conclude that Twist-mediated inhibition of apoptosis is accompanied by the repression of either the production or the accumulation of reactive oxygen species.

### Molecular mechanisms regulating the antioxidant activity of Twist

We next investigated the molecular mechanisms involved in the antioxidant activity of Twist. Because Twist proteins are transcription factors, we first tested whether they modulate the expression of the main cellular detoxifying enzymes. We quantified mRNA levels of Catalase, MnSOD, Cu/ZnSOD and Gpx1 by RT-qPCR in MEF cells transduced with Twist1, Twist2 or a control empty retroviral vector. As shown in Figure S4, none of the genes tested showed a significant differential expression in the presence of Twist. Similar results were obtained in HDF and REF52 (data not shown).

**Figure 5:**
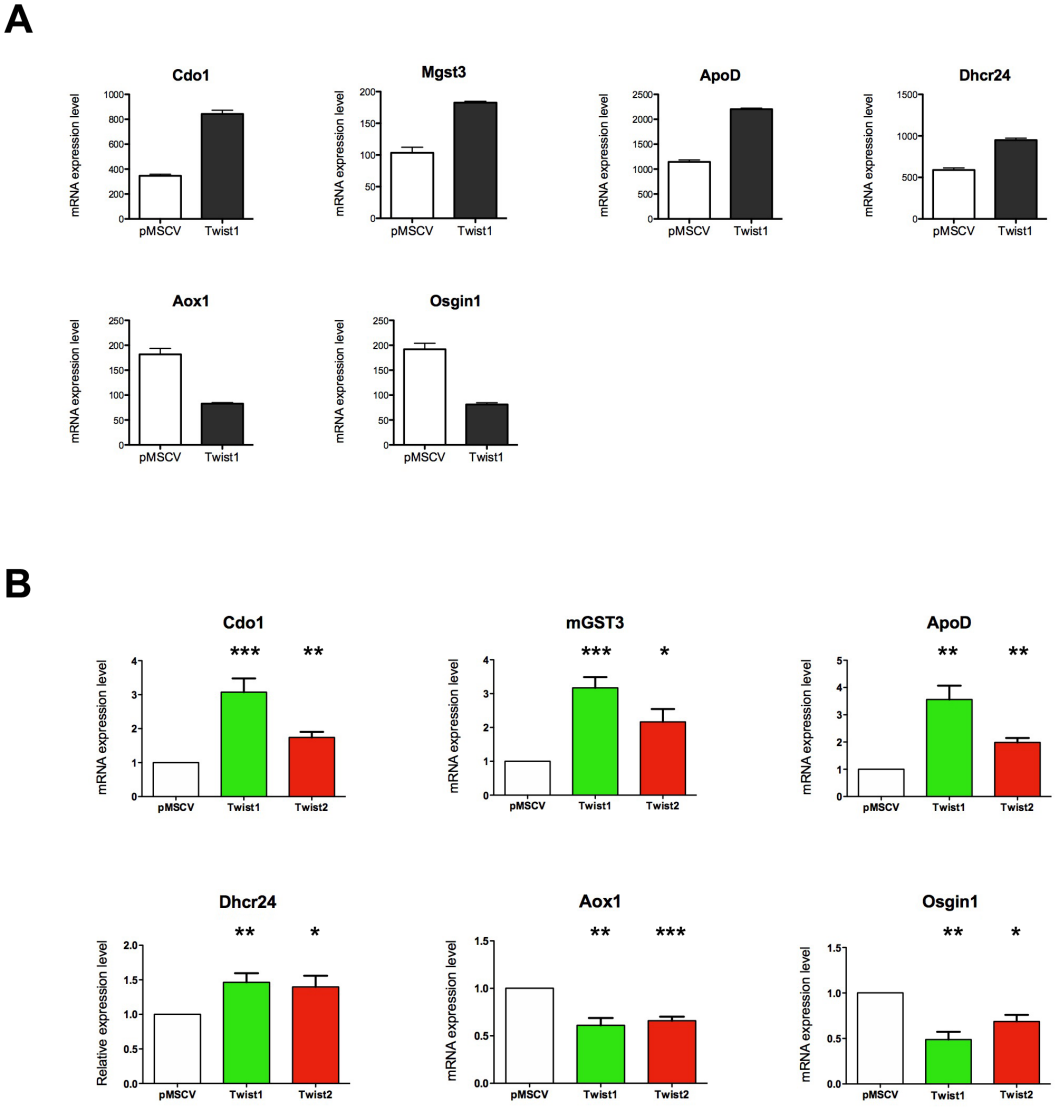
Molecular mechanisms regulating Twist antioxidant activity. (A) Microarray results for genes modulated by Twist1 and involved in the regulation of oxidative stress. Data show expression mRNA level of MEF-empty vector and MEF-Twist1. (B) mRNA levels for Cdo1, Mgst3 ApoD, Dhxr24, Aox1 and Osgin1 were assessed in MEF cells infected with empty pMSCV vector, pMSCV-Twist1 or pMSCV-Twist2. RNA levels were monitored by RT-qPCR, normalized with Hprt and adjusted relative to levels in pMSCV-transduced control cells.

In search of Twist target genes involved in this new activity, we next performed a microarray analysis comparing MEF cells expressing Twist1 with MEF transduced with an empty vector. 188 genes were found to be up-regulated and 246 repressed by more than 50% following Twist1 expression. The results confirmed that none of the main antioxidant enzymes were deregulated (data not shown). However, the analysis identified several genes, previously described as being involved in the control of oxidative stress, whose expression is regulated by Twist1 (Figure 5A). More precisely, we found four up-regulated genes: Cdo1, an enzyme required for the synthesis of taurine, a well known antioxidant compound [27], Mgst3, a microsomal gluthation S transferase carrying a peroxidase activity [28], ApoD, a protein involved in the inhibition of lipid peroxidation [29] and Dhcr24, described recently as a ROS scavenger [30]. Two genes were down-regulated: Aox1, an enzyme that produces ROS by oxidizing aldehydes [31] and Osgin1, a mitochondrial oxidative stress induced gene that was recently shown to increase intracellular ROS levels when overexpressed [32].

Because both Twist factors display an antioxidant activity, we next investigated whether the genes we identified were also modulated by Twist2. We generated MEF-empty vector, Twist1 and 2 lines and mRNA level of each target was quantified by RT-qPCR. As shown in Figure 5B, similarly to Twist1, 2 regulates the expression of the oxidative stress factors revealed by the microarray analysis. This result suggests that both Twist proteins modulate intracellular ROS level in a similar manner by controlling a common set of factors.

**Figure 6:**
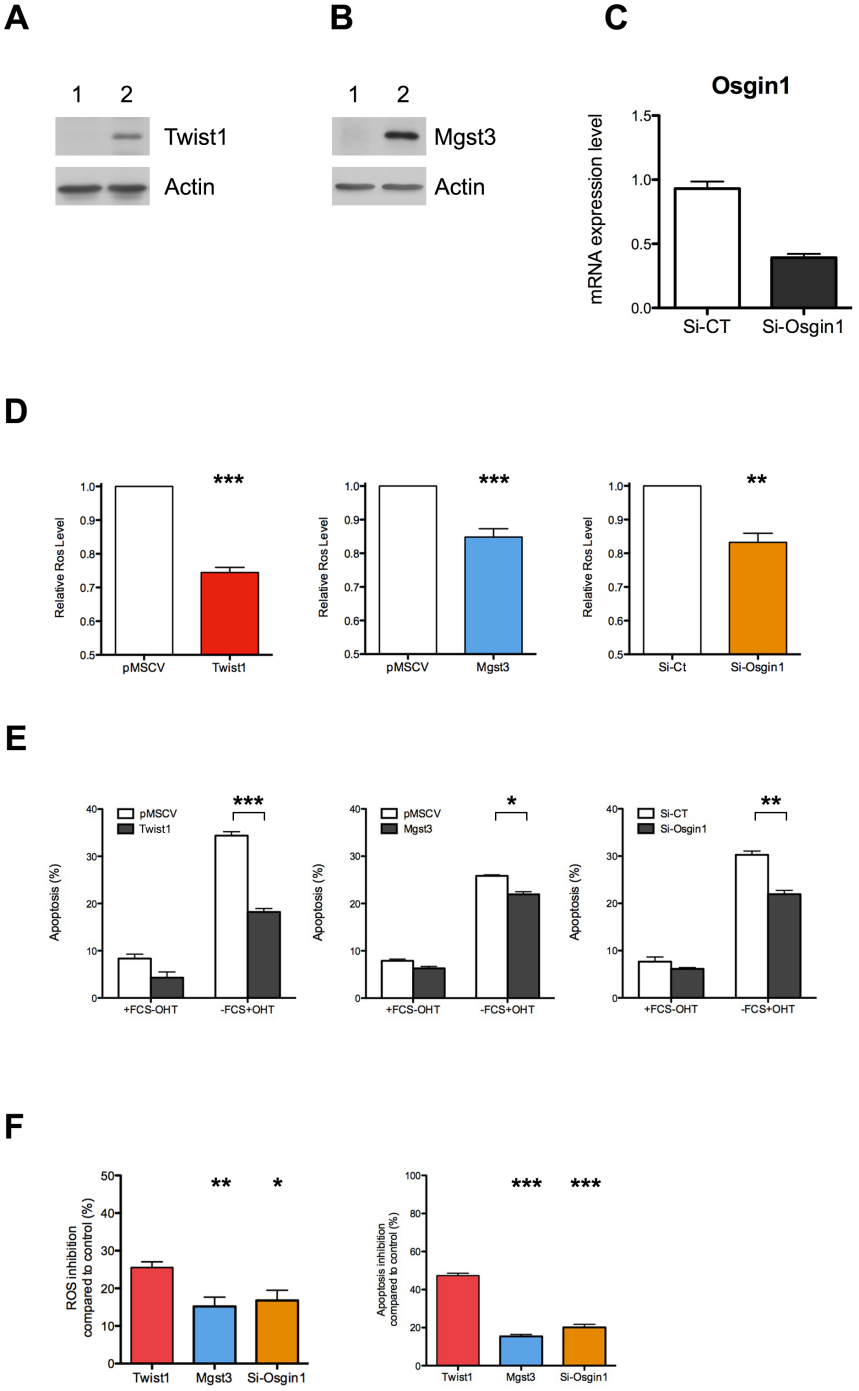
Role of Twist target genes in the control of ROS levels and apoptosis. (A, B) MEF cells were transduced with retroviruses encoding either Twist1 tagged with a Myc epitope (A), or with Mgst3 tagged with an HA epitope (B). Empty vector was used as a control. Expression of the transgenes was verified by western-blotting (1: Empty vector; 2: Transgene). (C) MEF cells were transfected with a siRNA control or a siRNA directed against Osgin1 and the silencing efficiency was measured by RT-qPCR normalized to HPRT. (D) Quantitative analysis of ROS levels of cell lines from A, B and C (*: n = 6, compared to control). (E) MEF-MycER cells were infected with either control (pMSCV), Twist1, or Mgst3 or were transfected with corresponding siRNA as in C (n = 3). Apoptosis was induced and determined as in Figure 4. (F) Alternative presentation of data from D and E, allowing better visualization of partial effects on ROS (*: n = 6, compared to red bar) and apoptosis (*: n = 3, compared to red bar) exercised by Mgst3 and Osgin-1. These graphs show the percentage of inhibition compared to control (pMSCV of si-CT).

Interestingly, none of the genes we identified was highly deregulated, suggesting that none by itself would account for Twist's antioxidant properties. We reasoned that an alternative mechanism of Twist's action might be a more global modulation of a genetic program of oxidative stress regulators. A similar situation has been reported for Nrf2, a master gene of intracellular ROS level control [33]. Upon induction of oxidative stress, this transcription factor is activated and triggers expression of numerous genes encoding enzymes involved in ROS detoxification [33]. It is the joint action of these proteins that efficiently removes excess of ROS from the cell. If such was indeed the mode of action of Twist, one would expect that modulation of individual target gene expression would only partially mimic Twist antioxidant activity. To test this hypothesis we overexpressed Mgst3, which is one of the genes up-regulated by Twist. In parallel, we silenced Osgin1, for which Twist is a repressor. Twist1 overexpressing MEF cells were used as a positive control. Ectopic expression and silencing were verified by western-blot and RT-qPCR, respectively (Figure 6A–C). Intracellular ROS levels were decreased similarly by up-regulation of Mgst3 or down-regulation of Osgin1 (Figure 6D). As expected, their antioxidant activity was lower than that displayed by Twist1 (Figure 6E), suggesting that each gene participates but does not fully substitute for Twist in decreasing ROS levels. Next, we tested the impact of the modulation of the same genes on c-Myc-induced apoptosis. As shown in Figure 6F, perturbation of the expression of Mgst3 and Osgin1 partially protects cells from cell death. Once again, this effect was not as marked as that produced by Twist1 (Figure 6G). We conclude that both Twist target genes are indeed involved in, but are not sufficient for, the Twist anti-apoptotic activity. Thus, our data indicate that Twist antioxidant and anti-apoptotic activities are mediated by a joint action of several of its downstream effectors.

In addition to their anti-apoptotic activity, Twist proteins have been very well described in terms of inducing EMT and enhancing cell motility [7]. We thus asked if the antioxidant and pro-migratory properties of Twist might be linked. We performed a wound-healing assay with cells expressing either Twist1 or Mgst3 or transduced with an empty vector as a control (Figure S5A and B). Whereas Twist expression accelerated wound closure, as expected, Mgst3 had no effect on cell migration in this assay. It would thus appear that lowering the intracellular ROS level has no significant influence on cell migration in this experimental model, suggesting that these two activities of Twist are uncoupled.

## Discussion

Twist transcription factors regulate several distinct cellular functions, [7]. Our study uncovered a novel activity carried by both Twist1 and Twist2, namely modulation of intracellular levels of reactive oxygen species (ROS). This finding might have important implications for understanding consequences of Twist activation in physiological and in physiopathological settings.

We concentrated our analysis on the impact of ROS on Twist's control of apoptosis. Twist displays anti-apoptotic activities in many cellular contexts and in response to multiple stimuli [7] and whether they are all governed by the regulation of ROS remains to be tested. For example, several reports have suggested that Twist expression in cancer cells is correlated with therapeutic drug resistance [16], [17] and it was shown that ROS are induced by therapeutic drugs and might participate in their cytotoxic action [34]. It is thus possible that the effect of Twist on drug resistance results, at least in part, from its antioxidant activity.

We demonstrated that the inhibition by Twist of c-Myc oncogene - induced apoptosis is correlated with its ability to lower ROS levels. This observation is especially interesting in the context of reports showing that mice invalidated for Twist1 display an increased apoptotic index during development [3]. Moreover, MEFs invalidated for Twist2 show an increased sensitivity to stimuli such as TNF-alpha [4], a cytokine that induces and requires ROS to exert its apoptotic activity. Thus, the antioxidant effect could participate in Twist regulation of cell death during development.

Modulation of ROS by Twist may also be important in adult tissues. It is well established that stem cells, in particular hematopoietic stem cells (HSC), display low levels of ROS [34]. This is essential for their maintenance and renewal, whereas an increase of ROS is required for differentiation [34]. The pattern of Twist expression is coherent with its role in regulation of this process. Specifically, Twist factors have been shown to control the maintenance of mesenchymal stem cells [5] and Twist2 inhibits differentiation of the myeloid lineage [6]. It will be of great interest to evaluate whether the antioxidant activity of Twist is conserved, and relevant, in these cellular settings.

Twist plays a major role in mediating metastasis [12]. In our experimental model Twist pro-migratory activity does not appear to be linked to its effect on the intracellular ROS levels. However, a recent study showed that Snail, another epithelial to mesenchymal transition inducing factor, triggers EMT and gives rise to a cancer stem cell phenotype by inhibiting ROS [35]. It is thus possible that Twist antioxidant activity, while not involved in the migration of mesenchymal cells, might act upstream during the transition process. These results also suggest that the control of intracellular ROS levels might be a common mechanism employed by different EMT-inducing factors. It will be of interest to test this hypothesis, notably in the context of the well-documented role of ROS in physiology and pathology and particularly in the process of cancer initiation and progression.

## Materials and Methods

### Ethics Statement

Experiments were performed according to national regulations and this study was specifically approved (ID approval N° D34-172-16) by the regional ethics committee of Languedoc-Roussillon (Comité Régional d’Ethique sur l’Expérimentation Animale- Languedoc-Roussillon), France.

### Constructs

N-terminal Myc-tagged Twist1 and Twist2 and HA-tagged Mgst3 murine cDNAs were cloned in pMSCV-puromycin retroviral vectors (Clontech). pBabe-MycER was a kind gift from Dr A. Gandarillas. ShRNA sequences against Twist2 were cloned into the previously described pRetro-SUPER vector [13]. An irrelevant sequence, with no significant hits when blasted against the NCBI nucleotide sequence NR database (5'-GTACGTACGATCACAGTCA-3') was used as an shRNA control.

### Cell culture

Human diploid fibroblasts (HDF) were a kind gift from Dr J. Piette [36]. Mouse embryonic fibroblasts (MEF), derived from C57BL/6 mice, were prepared from 13.5 DPC embryos. All cell lines including 293T cells [37] were cultured in Dulbecco’s modified Eagle medium containing 10% fetal bovine serum, 100 ng/mL streptomycin, and 100 U/mL penicillin at 37 °C with 5% CO2. HMEC were grown as recommended by the American Type Culture Collection (ATCC).

When appropriate, cells were treated with 1 µM 4-hydroxytamoxifen (Sigma Aldrich H7904), 2 mM Tiron (Sigma Aldrich 33724), 10 mM N-acetyl-L-cysteine (Sigma Aldrich A9165) or 20 µM Vitamin C (Ascorbic acid Sigma Aldrich A4403).

### Retroviral infection

1.5×10^6^ 293T cells were transfected twice at 24 hours intervals, with 3 µg of retroviral vector using jetPEI™ (Polyplus). Forty-eight hours after the last transfection, supernatants were collected, filtered (0.45 mm, Millipore), supplemented with 8 µg/ml of polybrene and added to cells to be transduced for 16 hr. M.O.I was always ≤ 1 as we noted that high viral titers induced a significant amount of non-specific oxidative stress. Infected cells were selected after 48 hr. with the appropriate antibiotics.

### Short Interfering RNA (siRNA) Transfection

Cells were seeded in 6-well plates 1 day before transfection. Cells were then transfected with 100 nM siRNA using Lipofectamine 2000 reagent (Invitrogen) according to the manufacturer's instructions. siRNAs were from Thermo Scientific except for siRNA against Osgin1, which was from IDT. Sequences are: siRNA Control: GUACGUACGAUCACAGUCA, siRNA TWIST2: siGENOME SMARTpool M-012862 siRNA Osgin1: MMC.RNAI.N027950.12.1.

### Immunoblot Analysis

Cells were lysed in boiling Laemmli buffer supplemented with protease inhibitors, then sonicated and complemented with DTT. Protein concentration was determined by BCA (Thermo Scientific) assay. Fifteen to twenty micrograms of total protein was loaded onto SDS-PAGE gels and transferred onto nitrocellulose membranes. The membrane was blocked with TBST (1× TBS with 0.1% Tween 20) + 5% milk at room temperature for 1 hr. and incubated with primary antibody and then with horseradish peroxidase (HRP)-coupled secondary antibody (Amersham). Activity was visualized by electrochemiluminescence. Antibodies used in this study are anti-Myc (9E10), anti-Actin (Sigma Aldrich) and anti-HA High Affinity (Roche).

### Reverse Transcription and Real-Time PCR

Total mRNA was isolated using a RNeasy mini kit (Qiagen). Reverse transcription was performed with random hexamers and M-MLV Reverse Transcriptase (Invitrogen). Real-time PCR was performed in triplicates with LC FastStart DNA Master SYBR Green I on a LightCycler rapid thermal cycler system (Roche Diagnostics), according to the manufacturer's instructions. Housekeeping genes GAPDH or HPRT were used for normalization. Primers sequences are listed in supplementary table (Table S1).

### ROS detection

Cells were incubated with 30 mM DHE (Invitrogen D11347) for 25 min or with 5 µM of CM-H_2_DCFDA (Invitrogen C6827) for 30 min, at 37°C in PBS. The cells were collected and fluorescence was quantified on a FACSCalibur flow cytometer using CellQuestPro software (BD Biosciences).

### Apoptosis analysis

Cells were plated at low density (10^5 ^cells/well) in 6 well plates 24 hours before induction of apoptosis. c-Myc-induced apoptosis was triggered by treatment with 1 µM 4-hydroxytamoxifen in 0.1% FBS. Apoptosis was monitored 24 hr. post-induction. To determine the percentage of apoptotic cells with externalized phosphatidylserine (PS), adherent and floating cells were collected and labeled with the Annexin V-Cy3 Apoptosis Detection Kit (Abcam) according to the manufacturer's instructions. Labeled cells were then analyzed on a FACSCalibur flow cytometer using CellQuestPro software (BD Biosciences).

### Microarray

mRNA purified from three different preparations of MEF-Empty vector and MEF-Twist1 were used for analysis. Microarray processing and subsequent analysis were performed by The Genomic Research Unit of the Luxembourg CRP (http://www.crp-sante.lu/Competence-centers/Luxembourg-Biomedical-Research-Resources/Genomics-Research-Unit). The full dataset analysis will be published in a future study (manuscript in preparation).

### Statistical analysis

Experiments were repeated at least three times. Data are presented as mean ± SEM. An Independent Student’s t test was performed to analyze the assay results; a two-tailed Student’s t test was used to compare the intergroup differences. Significance was accepted for values where P≤0.05 (*^/#^), P≤0.01 (**^/##^), P≤0.0005 (***^/###^).

## Supporting Information

Figure S1 **The antioxidant property of Twist is confirmed by DCFDA staining and is maintained in an epithelial cell line**. (A) ROS levels in the HDF cells used in Figures 1B and 1C were analyzed by flow cytometry following DCFDA staining and representative histograms are shown. (B) Quantitative analysis of ROS levels in HDF expressing either Twist1 or Twist2 relative to control HDF cells (*: n = 11, compared to white bar). (C) ROS measurements in HDF cells in the absence or presence of 5 mM NAC. (D) Quantitative analysis of ROS levels in cells incubated with NAC (*: n = 4, compared to white bar). (E) ROS level analysis in HMEC-hTert cells expressing Twist1 or Twist2.
(TIF)

Figure S2 **Antioxidant treatment protects cells from oxidative stress induced by Twist2 depletion**. HDF cells expressing a shRNA directed against Twist2 were cultured with 10 mM NAC for 24 hours. ROS levels were analyzed by DHE staining.
(TIF)

Figure S3 **ROS increase is mainly induced by serum starvation.** (A-B) REF52-MycER expressing or not Twist1 were cultured in 10% FBS or 0.1% FBS in the presence or absence of 4-hydroxytamoxifen (OHT). (A) ROS levels were measured after 8 hours and quantified as in figure 3B (*: n = 4, Twist1 compared to pMSCV). (B) Apoptosis was determined as in Figure 3C (presented as mean ± SEM of three independent experiments).
(TIF)

Figure S4 **Molecular mechanisms regulating Twist antioxidant activity**. mRNA levels for MnSOD, Cu/znSOD, catalase and Gpx1 were assessed in MEF cells infected with empty pMSCV vector, pMSCV-Twist1 or pMSCV-Twist2. RNA levels were monitored by RT-qPCR, normalized with Hprt and adjusted relative to levels in pMSCV-transduced control cells (n = 4, ns: non significant).
(TIF)

Figure S5 **Twist antioxidant activity is not linked to enhanced migration.** 1×10^6^ MEF-pMSCV, MEF-Twist1 and MEF-Mgst3 were seeded on a six well plate and 24 hours later a wound was made with a pipet tip. Wound width was monitored at time 0 and 16 hours. Distance of the wound was measured using ImageJ software. (A) Representative pictures of the three cell lines at 0 and 16 hours. (B) Quantification of wound width (presented as mean ± SEM of three independent experiments. *: compared to control at 18 hr.).
(TIF)

Table S1 **Primer sequences.**
(DOC)

Text S1 (DOC)
