# Supplementary material for: Modulation of Oxidative Stress by Twist Oncoproteins
Source: PLoS One. 2013 Aug 13;8(8):e72490. doi: 10.1371/journal.pone.0072490 (PMC3742535; doi:10.1371/journal.pone.0072490)
Supplement: Table S1 — Primer sequences. (DOC) [file pone.0072490.s006.doc]

**Table S1**

| **Genes** | **Sequence forward** | **Sequence reverse** |
| --- | --- | --- |
| **Twist2 (human & mouse)** | GCAAGAAGTCGAGCGAAGAT | GCTCTGCAGCTCCTCGAA |
| **MnSOD (mouse)** | CAGACCTGCCTTACGACTATGG | CTCGGTGGCGTTGAGATTGTT |
| **Cu/ZnSOD (mouse)** | AACCAGTTGTGTTGTCAGGAC | CCACCATGTTTCTTAGAGTGAGG |
| **Catalase (mouse)** | AGCGACCAGATGAAGCAGTG | TCCGCTCTCTGTCAAAGTGTG |
| **Gpx1 (mouse)** | AGTCCACCGTGTATGCCTTCT | GAGACGCGACATTCTCAATGA |
| **Cdo1 (mouse)** | GTGGATCAAGGAAATGGGA | CTTGATCATCTCGTTGGA |
| **Mgst3 (mouse)** | ATGGCTGTCCTCTCTAAGGAG | CTCTACCTTGTACTTCTTGCGG |
| **ApoD (mouse)** | GAACGGAAACATCGAAGTGCT | CTGGCTCTGAGACGTTGCTC |
| **Dhcr24 (mouse)** | TGCGAGTCGGAAAGTACAAG | GCCAATGGAGTTCAGCAAAG |
| **Aox1 (mouse)** | AGTGATGATCTCGCGGTACAA | CTGGTGTTGCCTAAGCCTTCT |
| **Osgin1 (mouse)** | CGGTGACATCGCCCACTAC | GCTCGGACTTAGCCCACTC |
| **Hprt (mouse)** | GCAGTACAGCCCCAAAATGG | GGTCCTTTTCACCAGCAAGCT |
| **GAPDH (human)** | ACACCCACTCCTCCACCTTT | TCCACCACCCTGTTGCTGTA |
