# Supplementary material for: Modulation of Oxidative Stress by Twist Oncoproteins
Source: PLoS One. 2013 Aug 13;8(8):e72490. doi: 10.1371/journal.pone.0072490 (PMC3742535; doi:10.1371/journal.pone.0072490)
Supplement: Text S1 — (DOC) [file pone.0072490.s007.doc]

**Supporting Text**

**Supplementary Figure 1: The antioxidant property of Twist is confirmed by DCFDA staining and is maintained in an epithelial cell line**. (A) ROS levels in the HDF cells used in Figures 1B and 1C were analyzed by flow cytometry following DCFDA staining and representative histograms are shown. (B) Quantitative analysis of ROS levels in HDF expressing either Twist1 or Twist2 relative to control HDF cells (*: n=11, compared to white bar). (C) ROS measurements in HDF cells in the absence or presence of 5mM NAC. (D) Quantitative analysis of ROS levels in cells incubated with NAC (*: n=4, compared to white bar). (E) ROS level analysis in HMEC-hTert cells expressing Twist1 or Twist2.

**Supplementary Figure 2: Antioxidant treatment protects cells from oxidative stress induced by Twist2 depletion**. HDF cells expressing a shRNA directed against Twist2 were cultured with 10mM NAC for 24 hours. ROS levels were analyzed by DHE staining.

**Supplementary Figure 3: ROS increase is mainly induced by serum starvation.** (A-B) REF52-MycER expressing or not Twist1 were cultured in 10% FBS or 0.1% FBS in the presence or absence of 4-hydroxytamoxifen (OHT). (A) ROS levels were measured after 8 hours and quantified as in figure 3B (*: n=4, Twist1 compared to pMSCV). (B) Apoptosis was determined as in Figure 3C (presented as mean ± SEM of three independent experiments).

**Supplementary Figure 4: Molecular mechanisms regulating Twist antioxidant activity**. mRNA levels for MnSOD, Cu/znSOD, catalase and Gpx1 were assessed in MEF cells infected with empty pMSCV vector, pMSCV-Twist1 or pMSCV-Twist2. RNA levels were monitored by RT-qPCR, normalized with Hprt and adjusted relative to levels in pMSCV-transduced control cells (n=4, ns: non significant).

**Supplementary Figure 5: Twist antioxidant activity is not linked to enhanced migration.** 1x10^6^ MEF-pMSCV, MEF-Twist1 and MEF-Mgst3 were seeded on a six well plate and 24 hours later a wound was made with a pipet tip. Wound width was monitored at time 0 and 16 hours. Distance of the wound was measured using ImageJ software. (A) Representative pictures of the three cell lines at 0 and 16 hours. (B) Quantification of wound width (presented as mean ± SEM of three independent experiments. *: compared to control at 18 hr.)
